# Long-Term Improvement of Chronic Low-Grade Inflammation After Bariatric Surgery

**DOI:** 10.1007/s11695-021-05315-y

**Published:** 2021-03-05

**Authors:** Anne Lautenbach, Fabian Stoll, Oliver Mann, Philipp Busch, Tobias B. Huber, Heike Kielstein, Ina Bähr, Jens Aberle

**Affiliations:** 1 III. Department of Medicine, University Medical Center Hamburg-Eppendorf, Martinistr. 52, 20246 Hamburg, Germany; 2 Department of General, Visceral and Thoracic Surgery, University Medical Center Hamburg-Eppendorf, Hamburg, Germany; 3 Institute of Anatomy and Cell Biology, Medical Faculty of Martin Luther University Halle-Wittenberg, Halle (Saale), Germany

**Keywords:** Bariatric surgery, Inflammation, High-sensitive CRP, Obesity, Weight loss

## Abstract

**Purpose:**

Bariatric surgery (BS) was shown to improve inflammatory markers in previous short-term follow-up studies. The aim of the present study was to assess the long-term effects of BS on chronic low-grade inflammation markers related to severe obesity. Moreover, the meaning of the type of BS procedure as well as the remission of type 2 diabetes (T2D) for inflammatory status up to 4 years after BS was analyzed.

**Materials and Methods:**

In a retrospective cohort study including 163 patients at baseline, inflammatory and metabolic parameters were assessed at 4 time points: before surgery (baseline), 6 months after surgery (visit 1), 2 years after surgery (visit 2), and 4 years after surgery (visit 3). Univariate regression analysis was used to identify variables that were thought to determine change in inflammatory parameters.

**Results:**

CRP, hs-CRP, leucocytes, and ferritin significantly declined in the mid- and long-term according to the U-shaped curve of weight loss (*p*<0.001). Change in body mass index (BMI) at long-time follow-up showed a significant linear effect on change in leucocytes (*B*=0.082; *p*<0.001) and change in hs-CRP (*B*=0.03; *p*<0.05). There was a strong, positive correlation between T2D and hs-CRP at visit 2 (*r*_*s*_=0.195; *p*<0.05) and visit 3 (*r*_*s*_=0.36; *p*=0.001). With regard to type of surgery and gender, there were no significant differences in inflammatory parameters.

**Conclusion:**

BS is able to reduce obesity-related chronic low-grade inflammation up to 4 years after surgical intervention. The improvement in metaflammation is related to the change in BMI and remission of T2D in the long-term.

**Graphical abstract:**

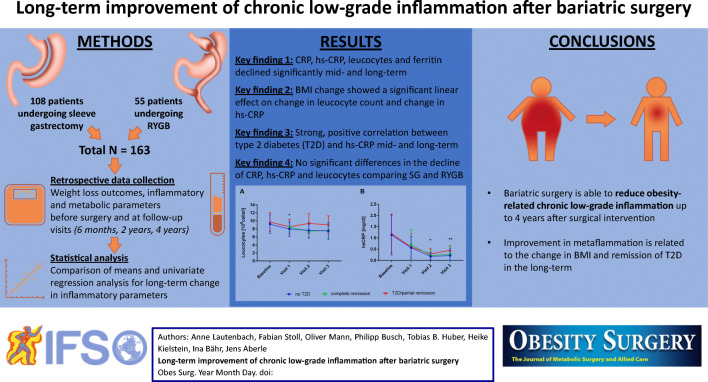

## Introduction

Obesity is associated with metaflammation, a metabolically triggered systemic chronic low-grade inflammation characterized by an augmented release of pro-inflammatory factors caused by the increased number of adipocytes and immune cells in adipose tissue [1]. Previous data demonstrated that the inflammatory condition in obesity contributes to the metabolic syndrome and other obesity-associated pathophysiological outcomes [2].

The inflammatory status in individuals with obesity can be measured by quantification of several inflammatory markers, like pro-inflammatory cytokines (e.g., tumor-necrosis factor-α and interleukin-6), number of leucocytes, and several adipokines. In addition, increased serum concentrations of the acute-phase reactants C-reactive proteins (CRP) and high-sensitivity CRP (hs-CRP) are commonly used to monitor inflammation and are strongly associated with metabolic syndrome, atherosclerotic cardiovascular disease, and T2D [3, 4]. Moreover, decreased levels of anti-inflammatory estrogens indicate inflammatory alterations in obesity [5].

Previous studies already demonstrated that loss of body weight and fat mass after bariatric surgery (BS) improves inflammatory parameters in patients with severe obesity [6–10]. However, in most of these studies inflammatory markers were analyzed after a follow-up duration up to 12 months after bariatric surgery, only 5 out of 116 studies had a follow-up period of more than 24 months according to a systematic review about the effect of BS on serum inflammatory markers of patients with obesity [11]. Moreover, only few data exist investigating the impact of surgery technique or gender on the postoperative inflammatory state. Therefore, the primary aim of the present study was to investigate changes of obesity-related inflammatory and metabolic parameters in male and female patients 6 month, 2 years, and 4 years after different bariatric procedures. Moreover, secondary aims were to examine whether the intensity of weight loss, the remission of T2D, and the type of surgery are associated with the grade of inflammation after BS.

## Materials and Methods

### Study Population

Male or female patients ≥ 18 years who underwent either sleeve gastrectomy (SG) or Roux-en-Y gastric bypass (RYGB) according to the S3 Leitlinie (Guidelines) *Chirurgie der Adipositas* [12] were included in the analysis. Patients with second step procedures during follow-up were considered having SG (*n*=6). Patients attended our obesity outpatient clinic between 2014 and 2020, which is certified as center of excellence for obesity and metabolic surgery by the European Accreditation Council for Bariatric Surgery. Exclusion criteria included incomplete records, history of acute inflammation (pulmonary, gastrointestinal, urogenital, cutaneous, or history of chronic autoinflammatory disease), and pregnancy. Of an initial population of 237 patients, 74 were excluded (*n*=163).

### Study Design

Follow-up data were retrospectively collected from 163 patients at baseline. To provide reasonable comparability between the cases, the available data were allocated 3 “visits” by time in relation to the procedure. In addition to baseline data −5.43±4.17 (mean±SD) months before surgery, data from visit 1 (*n*=160) were analyzed 6 months after surgery, data from visit 2 (*n*=128) 2 years after surgery, and data from visit 3 (*n*=75) 4 years post-BS.

### Variables

Data on height (cm), weight (kg), body mass index (BMI; kg/m^2^), gender, age, leucocytes (10^3^/μl), CRP (mg/l), hs-CRP (mg/dl), hemoglobin (g/dl), transferrin (g/l), transferrin saturation (%), ferritin (μg/l), estradiol (ng/dl), aspartate aminotransferase (AST; U/l), alanine aminotransferase (ALT; U/l), gamma-glutamyl transpeptidase (GGT; U/l), triglycerides (mg/dl), high-density lipoprotein cholesterol (HDL; mg/dl), low-density lipoprotein cholesterol (LDL; mg/dl), and HbA1c (%) were analyzed at baseline and during follow-up. Excess weight loss (EWL) in % was calculated by dividing the difference between initial BMI and final BMI by the difference between initial BMI and a target BMI of 25 kg/m^2^. Optimal weight loss was defined as losing at least 50% of the excess weight during the first 2 years after the procedure [13, 14].

Additionally, the prevalence of T2D was assessed. T2D was assumed if the diagnosis was pre-existing or if patients required antidiabetic medication or if HbA1c was above 6.5%. Complete remission of T2D was defined as HbA1c≤6.0% and no use of antidiabetic medication, partial remission of T2D was defined as HbA1c≤6.5% and no use of antidiabetic medication [15]. Due to the low number of cases, patients with partial remission were considered having T2D.

### Statistical Analysis

Independent variables were tested using Student’s *t*-test with Levene’s test for equality of variances. Normal distribution was tested with Shapiro-Wilk test.

Spearman’s rank correlation was performed to assess the relationship between glycemic control and inflammatory parameters. Linear regression analysis was performed to identify independent variables that determined the change in inflammatory parameters at long-term follow-up. The values are expressed as mean±SD. For all statistical tests, a *p*<0.05 was considered statistically significant. All analyses were conducted using SPSS software, version 27 for Windows, IBM, Germany.

## Results

### Demographic Data

Baseline characteristics are presented in Table 1. Mean age was 41.3±11.6 years, 75.4% of patients were female; 66.3% of patients underwent SG, and 33.7% underwent RYGB as initial weight loss procedure. Mean BMI was 51.63±8.02kg/m^2^; 30.7% of patients were diagnosed with T2D.

**Table 1:** Anthropometric and biochemical characteristics at baseline and follow-up visits

Parameter	Baseline	Visit 1	vs Baseline (*p*)	Visit 2	vs Baseline (*p*)	Visit 3	vs Baseline (*p*)
*N*	163	160		128		75	
Gender (F. *n*)	123	121		100		62	
Age (years)	41.3 ± 11.6	42.2 ± 11.6		44.4 ± 11.5		45.6 ± 10.6	
Initial treatment SG (*n*)	108	106		83		45	
Initial treatment RYGB (*n*)	55	54		45		30	
Second step SG to RYGB (*n*)	4	4		4		4	
Second step SG to SADI (*n*)	2	1		2		1	
Time to treatment (months)	−5.43 ± 4.17	5.32 ± 1.44		23.56 ± 3.94		44.67 ± 5.88	
Weight (kg)	153.5 ± 29.2	117.8 ± 24.6	<0.001	104.0 ± 26.1	<0.001	108.8 ± 31.9	<0.001
BMI (kg/m^2^)	51.63 ± 8.02	39.62 ± 7.22	<0.001	35.20 ± 7.99	<0.001	36.49 ± 9.67	<0.001
EWL (%)		47.3 ± 15.5	<0.001	63.8 ± 23.5	<0.001	58.1 ± 28.1	<0.001
Leucocytes (10^3^/μl)	9.3 ± 2.3	8.1 ± 1.8	<0.001	7.8 ± 2.0	<0.001	7.7 ± 2.1	<0.001
CRP (mg/l)	12.2 ± 8.4	7.5 ± 4.6	<0.001	5.2 ± 0.8	<0.001	5.5 ± 1.8	<0.001
Hs-CRP (mg/dl)	1.15 ± 0.88	0.58 ± 0.53	<0.001	0.20 ± 0.19	<0.001	0.25 ± 0.30	<0.001
Hb (g/dl)	13.9 ± 1.3	13.6 ± 1.2	0.024	13.4 ± 1.3	0.002	13.0 ± 1.7	<0.001
Tf (g/l)	2.9 ± 0.5	2.7 ± 0.4	<0.001	2.8 ± 0.5	0.038	2.9 ± 0.5	0.673
Tf-Sat (%)	17.4 ± 7.9	18.8 ± 9.3	0.123	21.0 ± 9.1	<0.001	17.2 ± 10.5	0.925
Ferritin (μg/l)	104.5 ± 93.5	105.8 ± 102.4	0.904	67.7 ± 82.3	<0.001	59.3 ± 89.8	<0.001
Estradiol-17beta (ng/dl)	53.8 ± 47.9	68.4 ± 75.9	0.040	83.8 ± 105.2	0.003	70.5 ± 97.9	0.165
AST (U/l)	25.0 ± 14.8	21.4 ± 9.1	0.008	18.8 ± 6.7	<0.001	19.8 ± 6.0	<0.001
ALT (U/L)	39.1 ± 26.2	25.9 ± 16.5	<0.001	25.3 ± 9.1	<0.001	27.2 ± 10.9	<0.001
GGT (U/l)	46.1 ± 59.2	26.8 ± 24.2	<0.001	24.3 ± 14.3	<0.001	25.7 ± 17.5	<0.001
Triglycerides (mg/dl)	228.1 ± 165.3	153.4 ± 75.1	<0.001	153.1 ± 124.3	<0.001	140.5 ± 94.6	<0.001
HDL-Cholesterol (mg/dl)	45.9 ± 13.7	47.8 ± 11.8	0.186	62.1 ± 15.9	<0.001	59.1 ± 15.8	<0.001
LDL-Cholesterol (mg/dl)	104.2 ± 32.9	103.5 ± 29.3	0.864	96.9 ± 30.9	0.063	104.6 ± 29.9	0.916
HbA1c (%)	5.86 ± 1.09	5.35 ± 0.71	<0.001	5.37 ± 0.76	<0.001	5.59 ± 0.83	0.036
HbA1c < 6.5% (%)	5.51 ± 0.44	5.22 ± 0.36	<0.001	5.21 ± 0.34	<0.001	5.42 ± 0.35	0.146
HbA1c ≥ 6.5% (%)	7.75 ± 1.52	7.74 ± 1.26	0.988	7.53 ± 1.37	0.370	7.94 ± 1.83	0.802

Data are reported as means±SD. *N*, number of individuals; *SG*, sleeve gastrectomy; *RYGB*, Roux-en-Y gastric bypass; *SADI*, single anastomosis duodeno-ileal bypass; *BMI*, body mass index; *EWL*, excess weight loss; *CRP*, C-reactive protein; *Hs-CRP*, high-sensitive C-reactive protein; *Hb*, hemoglobin; *Tf*, transferrin; *Tf-Sat*, transferrin saturation; *AST*, aspartate transaminase; *ALT*, alanine-aminotransferase; *GGT*, gamma-glutamyl transferase; *HDL*, high-density lipoprotein; *LDL*, low-density lipoprotein

### Weight Loss Outcomes

At visit 1, there were steep reductions in body weight (153.5±29.2 versus 117.8±24.6kg; *p*<0.001) and BMI (51.63±8.02 versus 39.62±7.22kg/m^2^; *p*<0.001). Further reductions in body weight and BMI occurred at visit 2 (104.0±26.1kg, *p*<0.001; 35.20±7.99kg/m^2^, *p*<0.001) and visit 3 (108.8±31.9kg, *p*<0.001; 36.49±9.67kg/m^2^, *p*<0.001) compared to baseline (Table 1). Weight regain at visit 3 was statistically not significant compared to visit 2 (*p*=0.251); 65.6% of patients showed a sustained weight loss of greater than 50% EWL during the first 2 years after surgery.

### Leucocyte count, CRP, and hs-CRP

Leucocyte count continuously dropped over the follow-up period (*p*<0.001 for all visits; Table 1). Weight loss was accompanied by a steep decline in mean CRP at visit 1 compared to baseline. Further reductions in mean CRP occurred at visit 2 and visit 3, respectively (*p*<0.001 for all visits). The slight increase in CRP at visit 3 was statistically not significant compared to visit 2 (*p*=0.114).

Accordingly, hs-CRP decreased from baseline to visit 1. At visit 2 and visit 3, a further decrease in hs-CRP was seen, respectively (*p*<0.001 for all visits). The slight increase in hs-CRP at visit 3 was statistically not significant compared to visit 2 (*p*=0.188).

At visit 1, 56.9% of patients showed complete normalization of inflammatory markers (leucocytes and hs-CRP). At visit 2 and visit 3, the percentage increased to 85.2% and 84.0%, respectively.

### Ferritin

At visit 1, ferritin levels remained stable compared to baseline. At visit 2 and visit 3, a significant decrease in ferritin levels compared to baseline was observed (*p*<0.001 for both visits). The decrease in ferritin levels at visit 3 was statistically not significant compared to visit 2 (*p*=0.501).

### Estradiol

Results demonstrate a significant increase in 17-ß estradiol at visit 1 and visit 2 compared to baseline (*p*<0.05 for both visits). At visit 3, estradiol levels were not significantly different from baseline, but increased by trend (Table 1).

### Glycemic Control

Mean HbA1c significantly decreased over the whole follow-up period compared to baseline (*p*<0.001 at visit 1 and visit 2, *p*<0.05 at visit 3). A gradual re-increase occurred at long-term follow-up, which was not statistically significant compared to visit 2 (*p*=0.057).

Accordingly, the percentage of patients with T2D decreased from 30.7 to 22.5% at visit 1 (*p*<0.001), 10.2% at visit 2 (*p*<0.001), and 6.7% at visit 3 (*p*<0.001). Thirty percent of patients achieved complete remission of T2D.

### Subgroup Analysis

#### Inflammatory Parameters and Estradiol Levels in Men and Women

**Fig. 1:**
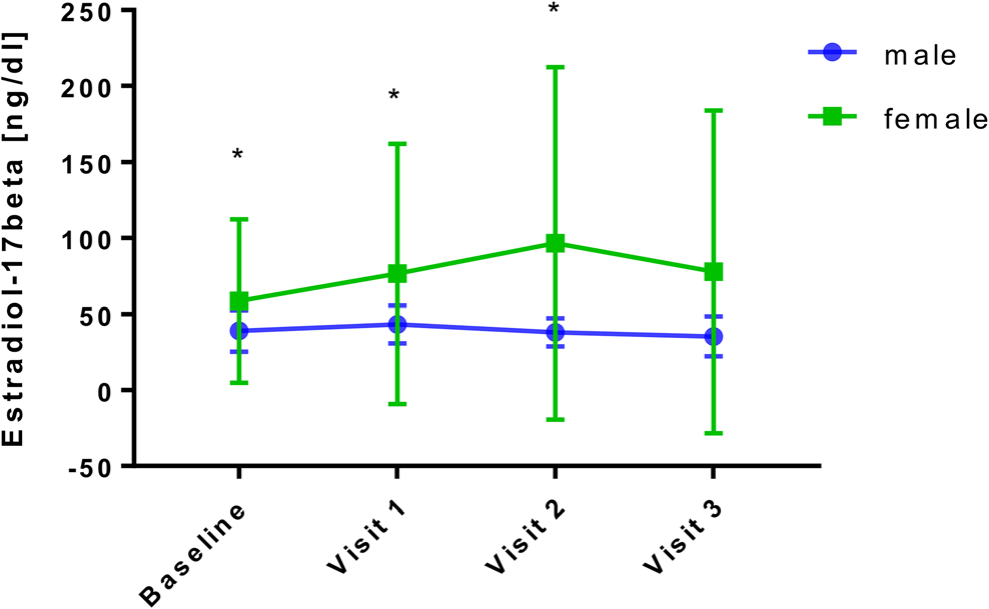
Baseline concentrations and postoperative course of 17ß-estradiol of male and female patients underwent bariatric surgery (BS). Baseline, before BS; visit 1, 6 months after BS; visit 2, 2 years after BS; visit 3, 4 years after BS. Data are presented as means±SD. *p<0.05

Leucocytes, CRP, and hs-CRP significantly dropped over the whole follow-up period in male and female patients (*p*<0.05). In both sexes, a significant decrease was observed in ferritin levels at visit 2 and visit 3, but not in the short-term (visit 1). At baseline (*p*<0.001), visit 1 (*p*<0.001), and visit 2 (*p*<0.05), ferritin levels in male patients were significantly higher than in female patients. In female patients, an increase in 17-ß estradiol by trend was observed, which was statistically significant at visit 2 (*p*<0.05). In male patients, a slight, but not significant, decrease in 17-ß estradiol by trend was detected (Fig. 1).

#### Inflammatory Parameters in Patients with SG vs. RYGB

Comparing SG and RYGB, CRP levels were significantly higher at visit 2 (*p*<0.05) in patients undergoing SG compared to RYGB. There were no significant differences in mean change from baseline of leucocyte count, CRP, and hs-CRP over the follow-up period between the surgical procedures. In the long-term, ferritin levels were significantly higher in patients undergoing SG compared to RYGB (*p*<0.05).

#### Inflammatory Parameters in Patients with T2D/Partial Remission Compared to Patients Without T2D/Complete Remission

**Fig. 2:**
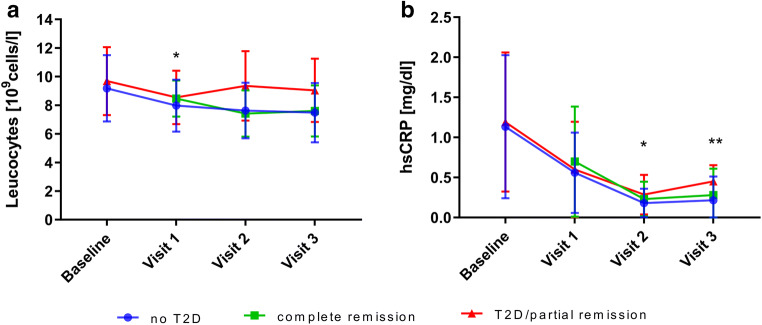
Baseline and postoperative course of leucocytes (**A**) and high-sensitive C-reactive protein (hs-CRP levels) (**B**) for patients without type 2 diabetes mellitus (T2D), complete remission of T2D and patients with T2D/partial remission. Hs-CRP, high-sensitive C-reactive protein. T2D, type 2 diabetes. Baseline, before BS; visit 1, 6 months after BS; visit 2, 2 years after BS; visit 3, 4 years after BS. Data are presented as means±SD. *p<0.05, **p<0.001, spearman’s rank correlation between hs-CRP/leucocytes and manifestation of T2DM

There was a strong, positive correlation between glycemic control and leucocyte levels at visit 1 (*r*_*s*_=0.16, *p*<0.05), hs-CRP at visit 2 (*r*_*s*_=0.195, *p*<0.05), and visit 3 (*r*_*s*_=0.36, *p*=0.001), which was statistically significant (Table 2, Fig. 2).

**Table 2:** Spearman’s rank correlation between glycemic control and inflammatory parameters

Parameter	Visit 1	Visit 2	Visit 3
Leucocytes (10^3^/μl)	**0.160 (*p*=0.043)**	0.119 (*p*=0.181)	0.179 (*p*=0.123)
CRP (mg/l)	0.026 (*p*=0.742)	0.031 (*p*=0.731)	0.019 (*p*=0.868)
Hs-CRP (mg/dl)	0.060 (*p*=0.454)	**0.195 (*p*=0.027)**	**0.364 (*p*=0.001)**
Ferritin (μg/l)	0.063 (*p*=0.429)	0.070 (*p*=0.430)	0.096 (*p*=0.412)
Estradiol-17beta (ng/dl)	0.089 (*p*=0.264)	0.100 (*p*=0.259)	0.004 (*p*=0.970)

*CRP*, C-reactive protein; *Hs-CRP*, high-sensitive C-reactive protein

#### Univariate Regression Analysis for Long-Term Change in Inflammatory Parameters

Change in BMI at long-term follow-up showed a significant linear effect on change in leucocytes (*B*= 0.082; *p*<0.001) and change in hs-CRP (*B*= 0.03; *p*<0.05). Results of univariate regression analysis are shown in Table 3. Patients with optimal weight loss showed a significant drop of leucocytes, CRP, and hs-CRP over the whole follow-up period (*p*<0.001). In patients with suboptimal weight loss, there was no significant decrease in leucocytes at long-term. In both subgroups, ferritin levels significantly decreased in patients with optimal (*p*<0.001) and suboptimal weight loss (*p*<0.05) in the long-term.

**Table 3:** Univariate regression analysis between change in BMI and change in inflammatory parameters at visit 3

Dependent parameter	B	SE (B)	*p*	*R*^*2*^
Δ Leucocytes (10^3^/μl)	0.082	0.023	<0.001	0.145
Δ CRP (mg/l)	0.192	0.103	0.065	0.046
Δ hs-CRP (mg/dl)	0.030	0.011	0.005	0.101
Δ Ferritin (μg/l)	0.204	0.842	0.809	0.001
Δ Estradiol-17beta (ng/dl)	−2.079	1.471	0.162	0.027

*CRP*, C-reactive protein; *Hs-CRP*, high-sensitive C-reactive protein; *B* regression coefficient. *R*^*2*^, R squared; *SE*, standard error; *CRP*, C-reactive protein; *Hs-CRP*, high-sensitive C-reactive protein

## Conclusion

Results of the present study provide important data on the long-term effects of BS on inflammatory markers. Since long-lasting inflammation in obesity is discussed as one of the main factors contributing to severe complications of viral infections, e.g., SARS-CoV-2, surgery-induced long-term changes in chronic low-grade inflammation are of particular clinical relevance [16].

In the present study cohort, a decline in CRP, hs-CRP, and leucocyte count was observed according to the U-shaped curve of weight loss with the lowest level of inflammation 2 years after BS followed by a gradual increase 4 years after BS. Assumingly, as a consequence of a gradual re-increase of food intake and body weight, metabolic parameters like LDL-cholesterol, liver enzymes, and HbA1c displayed the same U-shaped curve. However, 4 years following BS, complete normalization of hs-CRP occurred in 84.0 % of patients.

Moreover, the change in inflammatory parameters was related to the degree of weight loss. The most pronounced decline in inflammatory parameters was observed during the first 6 months following BS. Previous studies have observed similar short-term results [17–19]. At long-term follow-up, change in BMI showed a significant linear effect on change in leucocytes and change in hs-CRP. Patients with suboptimal weight loss showed increased leucocytes and hs-CRP levels in the long term. A recent study using latent class trajectory analysis demonstrated that steeper weight gain trajectories were associated with elevated risk of inflammation compared to more moderate weight gain trajectories, even after adjustment for initial weight, over a period of 18 years [20]. Analogously, our results clearly demonstrate that steeper weight loss was associated with decreased risk of inflammation in the long-term.

However, comparing SG and RYGB, there were no significant differences in the decline of CRP, hs-CRP, and leucocytes, even though EWL was significantly higher in patients undergoing RYGB 2 and 4 years post BS compared to SG*.* These data are in line with previous studies with a study duration up to 12 months [10, 21]. In contrast, a significant effect of type of surgery on CRP levels except the biliopancreatic diversion (BPD) was found according to a systematic review including studies with a study duration up to 24 months [11]. The lack of difference in our cohort might be explained by the fact that change in BMI, which must be considered the main driver for change in inflammatory parameters, was not significantly different in the long term between SG and RYGB. Moreover, the homogeneity of metabolic control between groups may play a crucial role. There were no significant differences in HbA1c in the long term, and HbA1c was only within the prediabetic range in both subgroups. Given the superiority of RYGB over SG in patients with obesity and T2D [22], it can be speculated that in a more metabolically unhealthy cohort, patients undergoing RYGB would have experienced a more pronounced decline in inflammatory parameters.

Patients with T2D showed increased inflammatory markers in the long-term (leucocytes and hs-CRP) compared to patients without T2D. In our cohort, 30.0% of patients achieved complete remission of T2D in the long term, whose profile was almost identical to those without T2D. Even though similar short-term results have been found showing that CRP showed a positive correlation with HbA1c≥6.5% [10], long-term results are of particular pathophysiological interest. T2D is known as a proinflammatory state with increased levels of hs-CRP and proatherogenic activity, which promotes increased micro- and macrovascular complications [23]. Data from 11 randomized controlled trials investigating clinical outcomes of metabolic surgery were not powered sufficiently to detect differences in micro- or macrovascular events, especially at relatively short follow-up [24]. However, our data highlight the clinical relevance of metabolic surgery, which can reverse and improve T2D, and therefore might prevent or slow atherogenesis in the long-term by breaking the vicious circle between inflammation and endothelial dysfunction [25].

There were no significant long-term differences in the decline of CRP, hs-CRP, and leucocytes between male and female patients. Different short-term results were found by Chiapetta et al. in a similar cohort, who observed a significant change in CRP only in female patients referring to the anti-inflammatory effects of endogenous estrogen [10, 26]. Weight loss restores gonadal dysfunction in men and women as soon as 6 months after surgery [27, 28], studies focusing on the long-term duration of restored gonadal function are lacking though. In female patients of our cohort, 17ß-estradiol levels increased by trend, but did not change significantly in the long term. In male patients, 17ß-estradiol decreased by trend, but there were no significant changes as typically seen following weight loss in male-obesity secondary hypogonadism [29, 30]. It can be assumed that more pronounced hormonal alterations after adjusting for age, gender, and BMI would have confirmed the anti-inflammatory properties of 17ß-estradiol [5]. Data from 6916 patients with severe obesity diagnosed with COVID-19 showed that mortality was most striking among men [31]. Therefore, studies addressing the impact of surgery-induced weight loss on sex hormone levels and inflammatory disease susceptibility are urgently needed.

Interestingly, results of the present study observed significantly increased ferritin levels in male patients at baseline and within 2 years of surgery, which may be explained by the higher BMI at baseline and throughout the study period that may lead to hyperferritinemia irrespective of body iron stores [32]. Moreover, the percentage of male patients with T2D (45%) was significantly higher compared to female patients (26%) which is a condition frequently associated with elevated levels of serum ferritin [33].

In conclusion, results of the present study clearly reveal a profound improvement in chronic inflammation up to 4 years following BS irrespective of type of surgery and gender. Close follow-up should be provided for patients to prevent gradual weight gain and associated worsening of inflammatory parameters in the long term.
